# Multiscale Porous Poly (Ether-Ether-Ketone) Structures Manufactured by Powder Bed Fusion Process

**DOI:** 10.1089/3dp.2021.0317

**Published:** 2024-02-15

**Authors:** Yaan Liu, Richard Davies, Nan Yi, Paul McCutchion, Binling Chen, Oana Ghita

**Affiliations:** Engineering, College of Engineering, Mathematics and Physical Sciences, University of Exeter, Exeter, United Kingdom.

**Keywords:** additive manufacturing, 3D printing, porous PEEK structures, powder bed fusion

## Abstract

The aim of the study is to create a multiscale highly porous poly (ether-ether-ketone) (PEEK) structure while maintaining mechanical performance; the distribution of pores being generated by the manufacturing process combined with a porogen leaching operation. Salt at 70 wt% concentration was used as a porogen in a dry blend with PEEK powder sintered in the powder bed fusion process. The printed porous PEEK structures were examined and evaluated by scanning electron microscopy, microcomputed tomography, and mechanical testing. The PEEK structures incorporating 70 wt% salt achieved 79–86% porosity, a compressive yield strength of 4.1 MPa, and a yield strain of ∼60%. Due to the salt leaching process, the PEEK porous frameworks were fabricated without the need to drastically reduce the process parameters (defined by the energy density [ED]), hence maintaining the structural integrity and good mechanical performance. The compression results highlighted that the performance is influenced by the printing orientation, level of the PEEK particle coalescence (controlled here by the ED), pore/cell wall thickness, and subsequently, the overall porosity framework. The porous printed PEEK structures could find potential uses in a wide range of applications from tissue engineering, filtration and separation to catalysts, drug release, and gas storage.

## Introduction

Porous polymers have drawn great attention because of their lightweight and high surface area allowing use in various applications such as gas storage and separation, filtration and separation membranes, catalysts, drug release, cell scaffold, and templates for structure replication.^1–6^ The choice of polymers depends on their properties and applications. Poly (ether-ether-ketone) (PEEK) is one of the highest performing engineering thermoplastic materials and is widely used in many fields, such as aerospace, automobile, and marine industries.^7–9^ There is an interest in producing porous PEEK structures due to the unique characteristics of the polymer: good resistance to chemicals, high resistance to operating temperatures up to 250°C, lightweight, and sterilization capability—opening the potential to be used for catalysts, membranes, and medical applications.^10,11^

A number of techniques have been developed for the fabrication of porous PEEK structures using sodium chloride as a porogen and using traditional processing methods such as compression molding, extrusion, and heat sintering.^12–16^ Phase separation methods have been reported to fabricate porous PEEK membranes by dissolving PEEK in solvents, followed by the extraction of PEEK from the solvent.^17,18^ Alternatively, a sulfonation treatment of PEEK was used as another way to obtain porous PEEK structures.^8,19^

Unfortunately, the traditional porous PEEK preparation methods are limited in design flexibility and customization.^1^ For this reason, Edwards and Werkmeister^20^ introduced two-dimensional (2D) PEEK scaffolds using weaving technologies and PEEK yarns. The scaffolds made using a multifilament had significantly smaller pores (80–100 μm) compared with monofilament scaffolds (261–280 μm). However, this technology is limited to two-dimensional fabrication.

Additive manufacturing (AM) or three-dimensional (3D) printing offers the ability to fabricate customized structures with complex geometries in 3D.^1,5^ Fused filament fabrication (FFF) is an AM method used extensively for the fabrication of scaffolds: plain PEEK^21,22^ or PEEK composites based on calcium hydroxyapatite (HA).^23–25^ Vaezi and Yang^26^ printed 3D porous PEEK samples with a minimum pore size of 150 μm by FFF. The average porosity was 14% and 31% by using 100% and 80% infill, respectively. Elhattab *et al.*^27^ generated porous PEEK with macropore sizes ranging from 800 to 1800 μm by adjusting FFF printing infill percentages from 70% to 50%. Although these studies revealed that the FFF technique provides reproducibility in printing porous PEEK scaffolds, the pores are controlled, identical, and the pore sizes are limited to a minimum of 100 μm.

Another AM technique, powder bed fusion (PBF) or laser sintering, which sinters polymeric powders into a 3D model layer by layer with a carbon dioxide laser, has been considered a promising method to fabricate PEEK structures as it can offer higher resolution.^28–30^ Several studies have been reported in fabricating porous PEEK structures by designing porous scaffold structures.^5,28,31,32^ Tan *et al.*^31,32^ created PEEK and PEEK/HA porous scaffolds by using different PBF process parameters, including different bed temperatures and laser powers. However, a very limited analysis of the porous structures has been presented. Microporosity, 73.5% ± 3%, was obtained when 10 wt% HA was added. It was assumed that higher porosity would be achieved when the laser power decreased from 18 to 16 W, but no tests have yet been done to prove the assumption.

Roskies *et al.*^5^ printed porous PEEK structures via PBF with relatively low porosity 36.38% ± 6.66%. All the above PBF studies rely on changes in energy density (ED) and particle coalescence in achieving porosity. In general, as the ED is decreased to increase the porosity, the mechanical performance is significantly reduced, and therefore, the method has limitations in levels of porosity if the mechanical integrity is to be maintained.

In this work, the porous PEEK was fabricated by PBF without drastically reducing ED to achieve high porosity. The article combines the accepted method of using a porogen material (in this case salt) with the PBF process to maximize the design freedom, to enhance and improve the porosity by widening the ranges of pore sizes while maintaining a good particle coalescence and good mechanical performance. The pore size and distribution of the porous structures as a function of orientation and PBF process parameters have been characterized and their mechanical properties have been analyzed.

## Materials and Methods

### Material preparation

PEEK 450PF (Victrex Plc, UK) powder (D_50_ = 50 μm) was used for the manufacture of PEEK components. The melting temperature of PEEK 450PF is 343°C, and the glass transition temperature is 143°C. To improve the powder flow, a heat treatment was carried out at 250°C for 24 h in an air-ventilated oven.^30^ The powder was then cooled down to room temperature naturally and sieved. After sieving, the powder was left resting for a day to avoid electrostatic charging. The powder with heat treatment is named as treated PEEK. Unrefined sea salt (Suma, UK) was milled using a powder grinder (Biolomix 700, UK) and sieved below 125 μm.

In Tan *et al.*'s work,^31^ 73.5% microporosity was achieved when 10 wt% HA was added. To obtain comparable or higher porosity in this work, 70 wt% of salt was added. The sieved salt was mixed with PEEK powder (PEEK and treated PEEK) by a roller mixer for 7 h at 30/70 PEEK: salt by weight ratio. The mixed powder-treated PEEK/salt (30/70) was dried at 100°C for 24 h in an air-ventilated oven before PBF and any characterization.

### Fabrication of porous PEEK by PBF

Previous studies have shown that thermally treated PEEK powder flows better than untreated PEEK,^30^ and therefore, treated PEEK/salt (30/70) was chosen as the powder for PBF. The PBF process was carried out using the EOSINT P 800 system (EOS, Germany). The details of the system including build chamber modes and the definition of processing parameters are described in detail in the previous publications.^30^

**FIG. 1.**
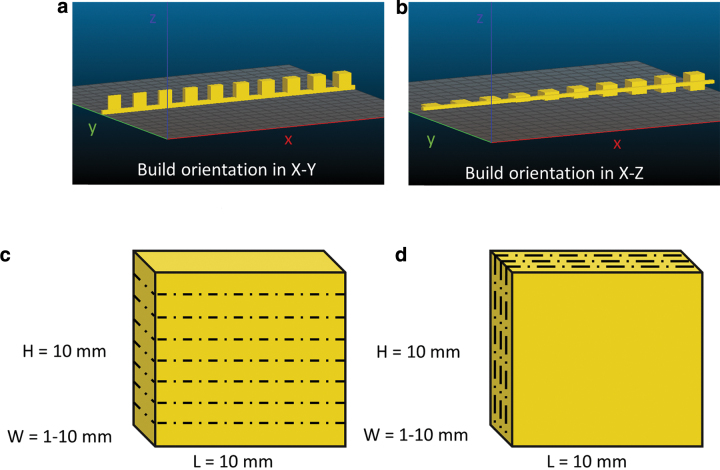
Samples with dimensions from L × W × H 10 × 1 × 10 to 10 × 10 × 10 mm with an increase of 1 mm in width were built in two different orientations **(a)** and **(c)** X–Y and **(b)** and **(d)** X–Z; *dash lines*: printing layers. *Dotted lines* indicate the layer deposition. Color images are available online.

Ten solid cuboids with constant length and height, but variable width, from 10 × 1 × 10 mm (L × W × H) to 10 × 10 × 10 mm with an increase of 1 mm in width were built in two different orientations X–Y (Fig. 1a, c) and X–Z (Fig. 1b, d) and five different laser powers (12, 13.5, 15, 16.5, and 18 W). All the samples with different width were built on a (200 × 2 × 4 mm) PEEK rod as a support structure that made printed structures easily found after the PBF process. All the processing parameters and corresponding ED are listed in Table 1. ED is the laser energy applied for sintering the materials and it is defined as^30^:

**Table 1.** Processing Parameters and Corresponding Energy Density

	Laser power (W)	ED (J/mm)	Laser speed (mm/s)	Hatching distance (mm)
Build 1	12.0	0.024	2550	0.2
Build 2	13.5	0.026	2550	0.2
Build 3	15.0	0.029	2550	0.2
Build 4	16.5	0.032	2550	0.2
Build 5	18.0	0.035	2550	0.2

ED, energy density.

$$ED=P∕\left(v\cdot SS\right)$$

Where *P* is laser power, *v* is laser speed, and SS is hatching distance. Laser speed (*v*) and hatching distance (SS) were kept constant at 2550 mm/s and 0.2 mm, respectively, with a layer thickness of 0.12 mm. Compression and 3-point bending test specimens were also built according to ISO 604 and ISO 178 standards in two orientations: X–Y and X–Z and the same laser powers (12–18 W) as previously used for building the cuboids.

After the PBF process, the printed samples were removed from the PEEK rod and transferred into a 2000 mL beaker containing water. The samples were magnetically stirred for 24 h to remove the salt. Once the salt leaching process finished, the porous structures were placed in an oven at 100°C for 24 h to dry. The full removal of the salt was confirmed by the microcomputed tomography (CT) data, an indication that the porous structure includes interconnected pores.

### Scanning electron microscopy and energy dispersive spectrometer characterization

Scanning electron microscopy (SEM) images were obtained by a Tescan VEGA3 SEM (Tescan, UK). Powders and printed porous structures were spread or pasted on conductive carbon tape and then sputter-coated with 15 nm of Cr in case of accumulation of electrostatic charge. The secondary electron imaging was carried out using an accelerating voltage of 20 kV. An X-max energy dispersive spectrometer detector (Oxford Instrument, UK) was used to obtain element mapping.

### Micro-CT

To assess the porous structures, the largest cuboid samples (10 × 10 × 10 mm) were quantitatively analyzed using a micro-CT (VERSA XRM-500; Zeiss, Germany) under scanning conditions of 50 kV and 80 μA. A high resolution of 2.03 μm per voxel was achieved. All micro-CT results were analyzed using the Avizo-9.0.1 software to obtain the porosity, volume of the PEEK frameworks, and salt residues. Three 500 × 500 × 500 μm cubic structures were extracted (using the exact subvolume function) as regions of interest for each cuboid sample to get the average data, and the images were then processed and improved by using a range of functions and settings such as the following: nonlocal means filter deblur, unsharp masking functions.

**FIG. 2.**
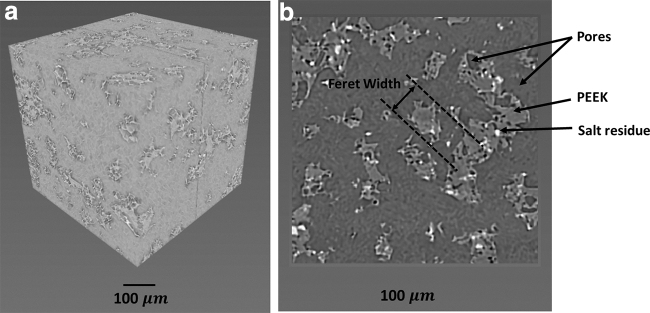
**(a)** 3D micro-CT scan of treated PEEK/salt (30/70)-12 W built in X–Y; **(b)** 2D cross section (Y–Z) of micro-CT scan of treated PEEK/salt (30/70)-12 W built in X–Y showing the three phases: *white* represents the salt residue, the *dark gray* represents the pores, and the *lighter gray* the PEEK structure, and Feret width of PEEK structures in 2D projection from 3D object. 2D, two dimensional; 3D, three dimensional; CT, computed tomography; PEEK, poly (ether-ether-ketone).

The three phases within the structure: the pores, the PEEK framework, and salt residue were separated by the threshold tool and the average volume fractions were calculated by the software (Fig. 2). To measure the wall thickness of the PEEK framework, the separated PEEK regions were measured by Feret width 3D function (Avizo-9.0.1—PEEK Label—Label Analysis—Feret—Width 3D—Interpretation 3D). Feret width is the minimal distance between parallel tangents of the PEEK structure (illustrated in Fig. 2b). Using the 3D interpretation mode of the software, the Feret width 3D function allows measuring of 20,000–50,000 Feret width values for a specific PEEK framework (31 directions by default). The wall thickness distributions were calculated and plotted.

### Mechanical testing

Compression test specimens with a dimension of 10 × 10 × 4 mm were built in two orientations: X–Y and X–Z with the five laser powers (12–18 W). Compression tests were performed by using the Lloyd LR3000k Universal Materials Testing Machine (Lloyd Instruments, UK) according to the ISO 604 standard. The test speed was 1 mm/min. Three-point bending test specimens with a dimension of 80 × 10 × 4 mm were built in two orientations: X–Y and X–Z and the laser powers of 12, 15, and 18 W. Three-point bending tests were carried out by using a mechanical testing machine (LLOYD instrument EZ20, UK) according to the ISO 178 standard. The span size was 64 mm and the test speed was 5 mm/min. Three samples for each type of porous PEEK materials were tested and the average data were obtained.

### Statistical analyses

Statistical analyses were conducted using a one-way analysis of variance (ANOVA) with a *post hoc* Tukey test. The tests were performed using the SPSS (IBM SPSS version 26) software. A *p*-value of <0.05 represents a significant difference between compared groups.

## Results and Discussion

### Printed porous PEEK structures and SEM images

Porous PEEK structures were printed using the treated PEEK/salt (30/70) blend. To assess the printability on samples with different thicknesses, 10 cuboids with variable widths from 10 × 1 × 10 to 10 × 10 × 10 mm with an increase of 1 mm in width were built in two different orientations X–Y and X–Z. Five laser powers from 12, 13.5, 15, 16.5, and 18 W were used.

Supplementary Figure S1 shows the cuboids fabricated using a laser power of 18 W (treated PEEK/salt [30/70]-18 W). Sample weight dropped by ∼70% after salt leaching, suggesting that most of the salt particles were leached out due to connected pores. Except for the samples of 10 × 1 × 10 mm printed in the X–Y direction, all the other samples maintained their shape and structure after the salt leaching. The samples of 10 × 1 × 10 mm built in X–Y were too thin and porous to keep the structure after the PBF process. The weight and size changes before and after the salt leaching are listed in Supplementary Table S1.

**FIG. 3.**
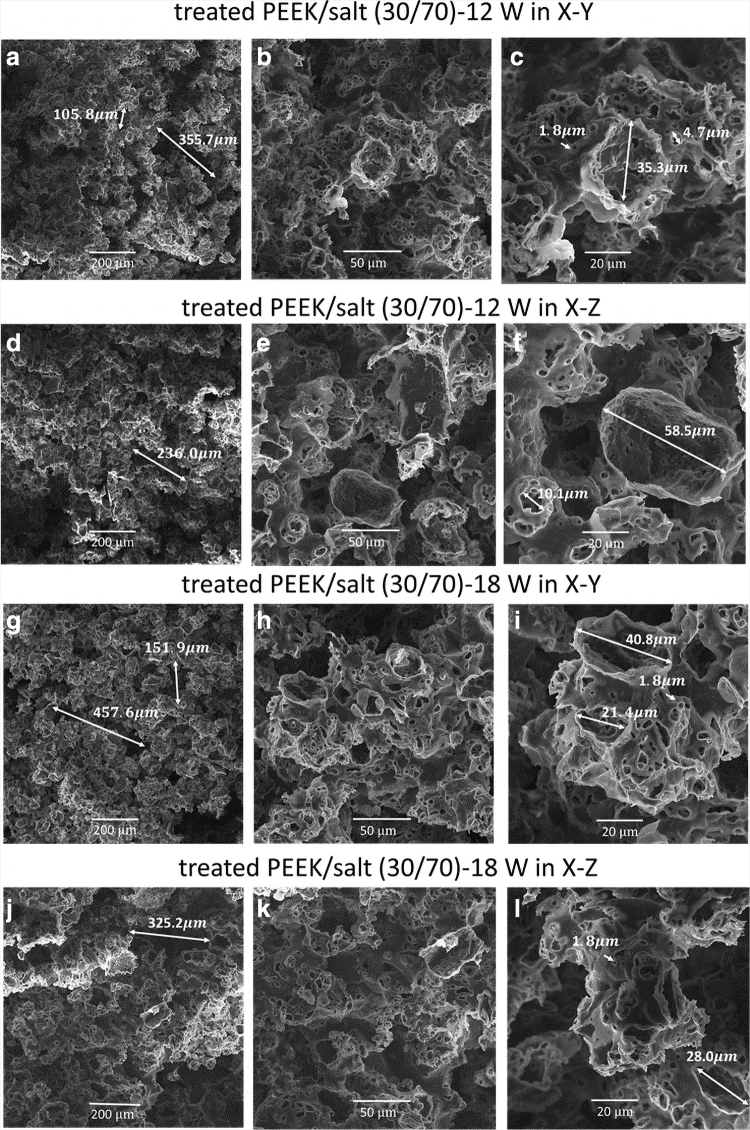
SEM images of the fractured surface, porous, of **(a–c)** treated PEEK/salt (30/70)-12 W in X–Y; **(d–f)** 12 W in X–Z; **(g–i)** 18 W in X–Y; and **(j–l)** 18 W in X–Z with different magnifications. SEM, scanning electron microscopy.

Figure 3 shows the fracture surfaces (3-point bending fractured samples) of treated PEEK/salt (30/70) fabricated at laser powers of 12 and 18 W in X–Y and X–Z directions. Some large connect pores around a few hundred microns were found in low-magnification images. In higher magnification images, round pores of ∼50 μm and smaller pores of a few microns were randomly generated after the salt leaching process. No obvious differences in pore sizes were observed when different laser powers were used and no obvious differences in orientations were found. It is difficult to make the distinction and differentiation between open and closed pores through the SEM images and the fact that most of the salt was leached out of the sample indicates a significant amount of interconnectivity.

### Micro-CT

**FIG. 4.**
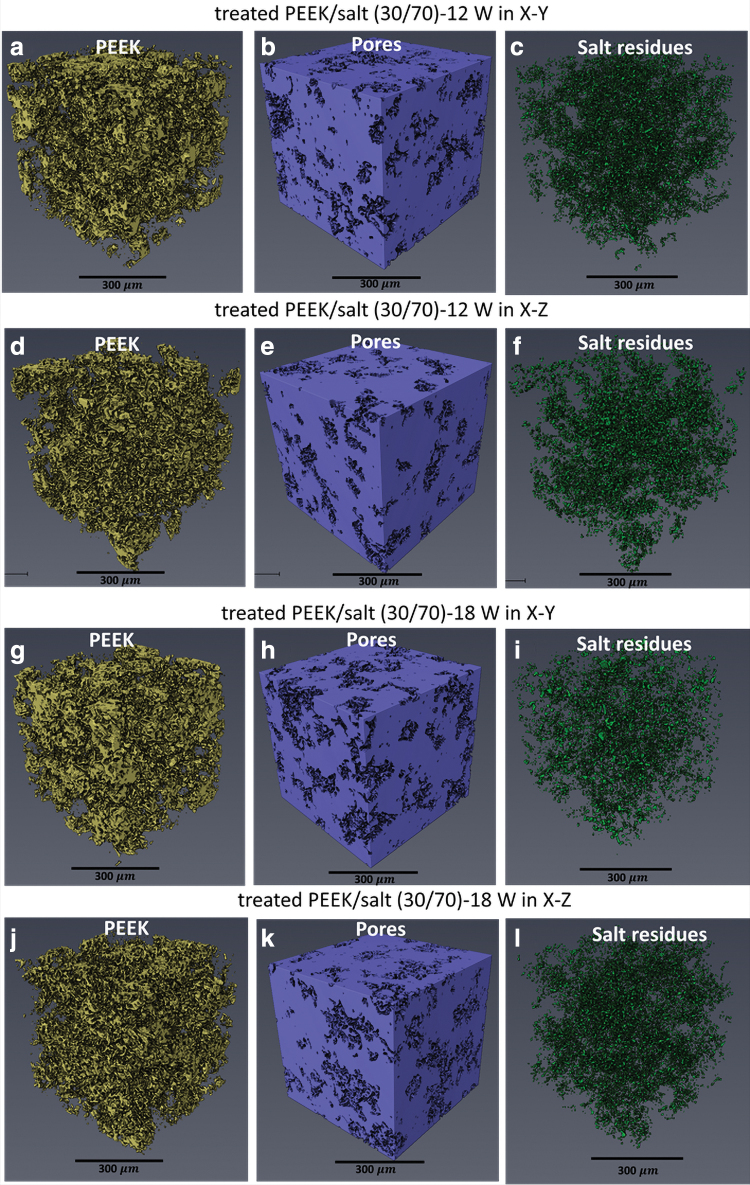
Micro-CT scans of PEEK, pores, and salt residues of **(a–c)** treated PEEK/salt (30/70)-12 W in X–Y; **(d–f)** 12 W in X–Z; **(g–i)** 18 W in X–Y; and **(j–l)** 18 W in X–Z. Color images are available online.

To better understand the porous structures, the largest cuboid sample (10 × 10 × 10 mm) fabricated using 12, 15, and 18 W in both X–Y and X–Z directions was quantitatively measured by micro-CT. Three phases were observed: PEEK framework, pores, and some salt residue. Images of treated PEEK/salt (30/70) printed at 12 and 18 W are shown in Figure 4, the volumes of pores being much higher than the PEEK frames and salt residues, suggesting highly porous structures.

The porosity, volumes of PEEK framework, and salt residues are calculated and summarized in Table 2, and details of the statistical analysis are shown in Table 3. In this work, the weight ratio of salt was 70 wt% and the calculated volume ratio was 58.8% (salt density is 2.16 g/mL, and PEEK density is 1.32 g/mL). If all the pores were generated by salt and all the salt particles were removed after the salt leaching process, the theoretical porosity should be 58.8%.

**Table 2.** The Porosity, Volume Fraction of Poly (Ether-Ether-Ketone) Frameworks, and Salt Residues

	PEEK framework %	Porosity %	Salt residue %
12 W in X–Y	15.5 ± 1.4	83.5 ± 0.1	1.1 ± 0.1
12 W in X–Z	13.3 ± 1.0	85.8 ± 1.1	1.1 ± 0.1
15 W in X–Y	14.4 ± 2.2	84.0 ± 2.5	1.6 ± 0.3
15 W in X–Z	14.8 ± 3.5	84.1 ± 2.3	1.1 ± 0.2
18 W in X–Y	19.7 ± 0.8	78.8 ± 0.5	1.5 ± 0.3
18 W in X–Z	19.1 ± 0.5	79.0 ± 0.5	1.9 ± 0.1

PEEK, poly (ether-ether-ketone).

**Table 3.** Analysis of Variance on Porosity Results

Groups	*p*	Results
12 W in X–Y		
12 W in X–Z	0.467	No significant difference
15 W in X–Y	0.999	No significant difference
15 W in X–Z	0.996	No significant difference
18 W in X–Y	0.020	Significant difference
18 W in X–Z	0.025	Significant difference
12 W in X–Z		
15 W in X–Y	0.646	No significant difference
15 W in X–Z	0.733	No significant difference
18 W in X–Y	0.001	Significant difference
18 W in X–Z	0.001	Significant difference
15 W in X–Y		
15 W in X–Z	1.000	No significant difference
18 W in X–Y	0.012	Significant difference
15 W in X–Z		
18 W in X–Z	0.014	Significant difference
18 W in X–Y	0.009	Significant difference
18 W in X–Z	0.011	Significant difference
18 W in X–Y		
18 W in X–Z	1.000	No significant difference

However, the samples built at 12, 15, and 18 W show a higher porosity than 58.8%, suggesting that the manufacturing process itself also generated pores. It is known that higher power and subsequently a higher ED leads to a denser part, whereas a lower ED reduces the level of particle coalescence, creating a more porous structure. This explains the lower porosity noticed in the higher density samples of 18 W.^30^ Statistical analysis results (Table 3) show that structures printed with 18 W are significantly different from the other powers applied.

### Mechanical properties

#### Compression test

**FIG. 5.**
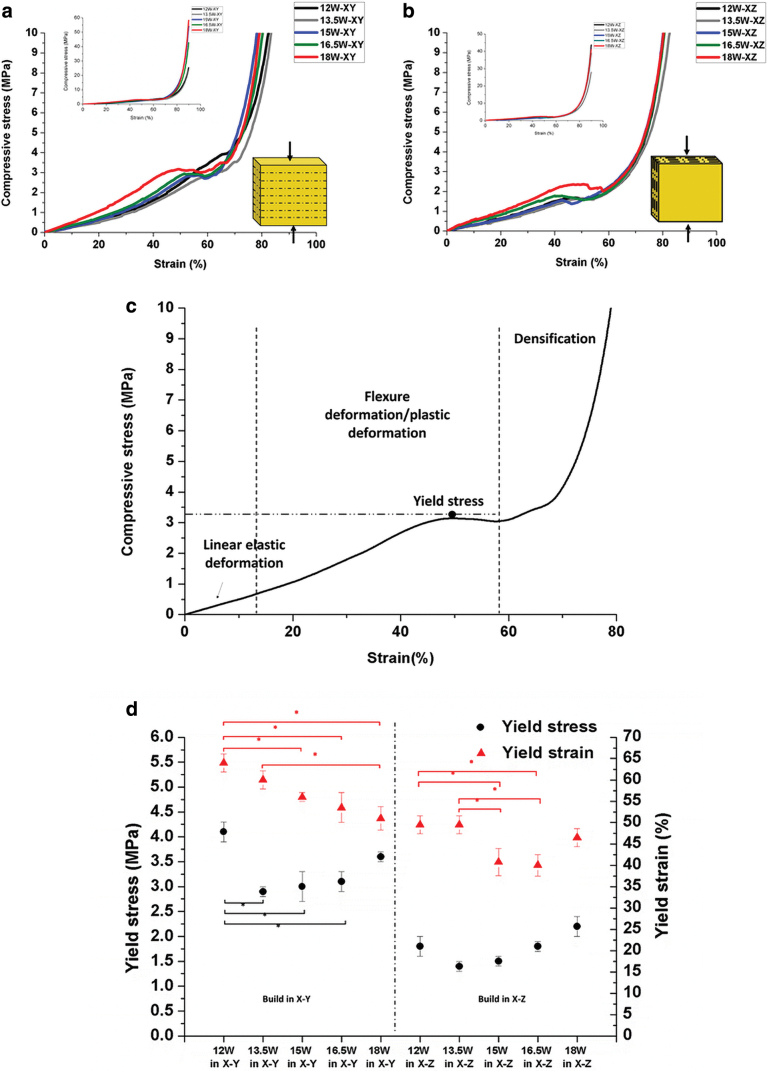
The typical compressive stress–strain curves of porous PEEK structures of treated PEEK/salt (30/70) fabricated by different laser powers and orientations: **(a)** in X–Y and **(b)** in X–Z; **(c)** compressive stress–strain curves of porous structures including three staged responses: linear elastic deformation, plastic yielding/flexure deformation and densification, and **(d)** compressive yield stress and strain of porous PEEK structures 450PF-T250_salt 70. *p < 0.05, indicating a significant difference between groups. Color images are available online.

Typical compressive stress–strain curves of printed porous structures PEEK/salt (30/70) in both orientations are shown in Figure 5a and b. The curves show the typical three-stage response as follows^33,34^: (1) linear elastic deformation at small strain controlled by cell wall bending; (2) yielding deformation with progressive buckling followed by a plateau for an elastoplastic material with failure of cell wall, and (3) densification at large strain (Fig. 5c). Yield stress or compressive stress is defined as the peak value in the plastic deformation region. Yield strain is the strain at yield stress. Because of the sample size and experimental set up, the strain in the linear elastic region was extremely sensitive to small displacement and therefore difficult to evaluate for calculating the compressive modulus.

However, the strain at yield point was clearly defined in the original test, and therefore, the compressive yield stress and strain are calculated and presented in Figure 5d. The compressive yield stress of the X–Y samples varied in the range 3–4 MPa, whereas the yield stress of the X–Z samples ranged between 1 and 2 MPa.

An interesting study was carried out by Zheng *et al.*,^25^ printing PEEK-HA scaffolds of different pore sizes (0.2–2 mm) using FFF technology. In their study, the compressive strength of the scaffolds along the Z compressive direction was higher than that of the scaffolds tested along the X direction, for the scaffolds of the same pore size, and the compressive strength decreased dramatically from 35 to 2 MPa as the pore size increased from 0.2 to 2 mm. The compressive strength of scaffolds of 1.6 mm pore size achieved ∼2 to 5 MPa, in the same range as our compressive yield stress values, indicating that the samples created here are possibly more sensitive to the larger pore sizes.

Unfortunately, the results of Zheng *et al.*^25^ cannot be directly used here for comparison as the samples achieved through our study have a wide pore size distribution and not a controlled single size, as is the case of FFF-printed scaffolds.

It was found that printing orientation affects compressive stress significantly. The yield stress of the samples printed in X–Y is higher than that in X–Z. For the samples printed in X–Z orientation, the load direction was parallel to the layer–layer interface. As the layer to layer bonding is always the weaker direction of most AM processes, the load applied tends to break layers apart easier, the fracture leading to lower stress.

For both orientations, the yield stress initially decreased relative to the lower laser power used (12 and 13.5 W) and then increased for higher laser power (13.5–18 W). Although the samples printed at 12 and 15 W had similar porosities, the 12 W showed higher yield stress and strain than 15 W (Fig. 5). The samples printed at 12 W showed a very short plateau region with less failure of cell wall, but presented cell wall bending deformation followed by cell densification (curves in Fig. 5).

The literature shows that the elastic deformation of foam structures before yielding points is controlled by cell wall bending, which is related to the structure density and cell geometry such as cell sizes, cell shapes, and cell wall thickness.^34^ The compressive yield stress of microcellular polystyrene foams prepared using a carbon dioxide foaming agent with approximately constant densities increased with increasing wall thickness.^34^ The compressive yield stress is proportional to $t^{4}h^{-2}I^{-2}$, where *t* is cell wall thickness, *h* is cell height, and *l* is cell width.

**FIG. 6.**
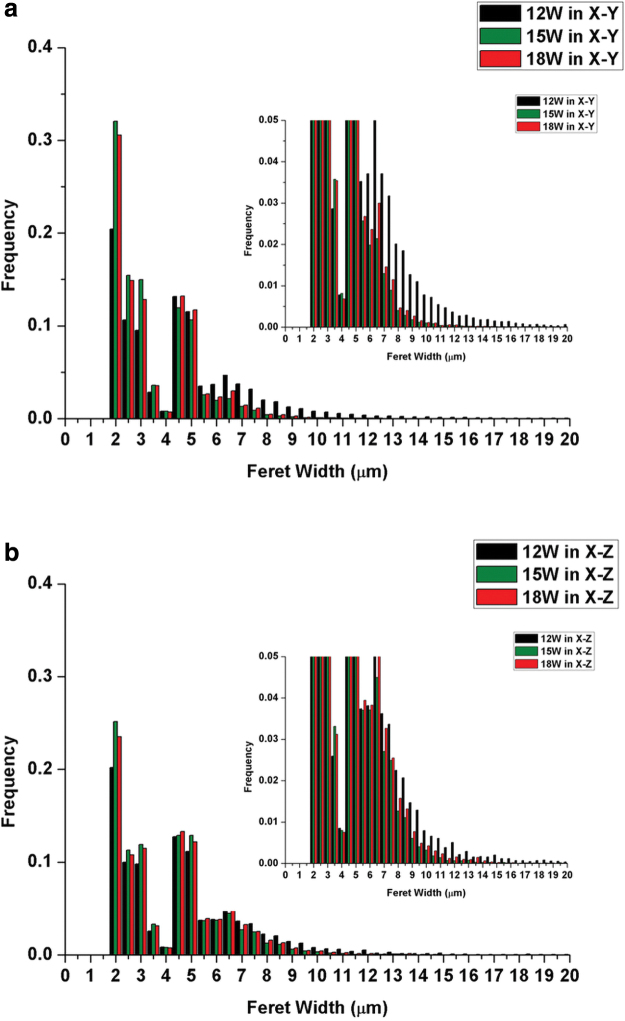
Feret width (PEEK wall thickness) distributions of PEEK framework-treated PEEK/salt (30/70) measured by micro-CT **(a)** 12, 15, and 18 W in X–Y; **(b)** 12, 15, and 18 W in X–Z. Color images are available online.

In our study, the differences in yield stress between the samples printed at 12 and 15 W may be due to different wall thicknesses within the PEEK framework. The Feret width of PEEK frameworks measured by micro-CT was used to measure PEEK wall thickness, and Figure 6 shows the Feret width distributions of PEEK frameworks. The porous samples printed at 12 W in X–Y had a significant increase in wall thickness in the region between 6 and 20 μm. In comparison with the 15 W laser power, the 12 W power will create less melt, less flow around the salt particles, and less coalescence. An increased energy will lead to better melt flow, which although desirable in most of the cases, here it will create lower wall thickness. This is most likely the reason the yield stress of 12 W is higher than 15 W for the X–Y orientation.

The samples printed in X–Z using 12 and 15 W show similar wall thickness distributions and similar yield stress values. The ANOVA also confirms that there is a significant difference in yield stress between 12 and 15 W in X–Y orientation, while no significant difference between 12 and 15 W in the X–Z orientation.

In this study, the porosity shows less effect on compressive yield stress compared with building orientation and wall thickness. The samples printed at 18 W have lower porosity than 15 W (∼79% and ∼95%, respectively), but similar wall thickness distributions in both X–Y and X–Z orientations. The yield stress at 18 W increased slightly compared with 15 W, but the ANOVA suggests no significant differences.

#### Three-point bending test

**FIG. 7.**
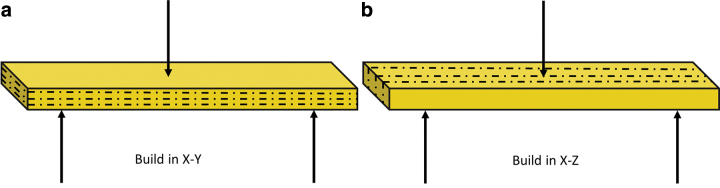
Samples built in **(a)** X–Y and **(b)** X–Z for 3-point bending test. Color images are available online.

**FIG. 8.**
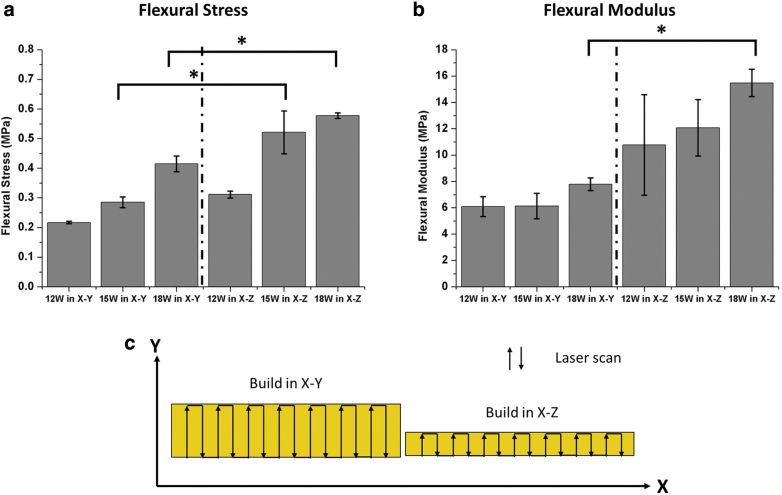
**(a)** Flexural stress and **(b)** modulus of porous PEEK structures. *p < 0.05, indicating a significant difference between printing orientations X–Y and X–Z using the same laser power, and **(c)** laser scan vectors (Y direction) of samples printed in X–Y and X–Z for 3-point bending samples. Color images are available online.

Figure 7 illustrates the 3-point bending test performed using the porous PEEK printed in X–Y and X–Z orientations. The flexural stress (defined as maximum stress) and flexural modulus of the porous PEEK structures printed at 12, 15, and 18 W are plotted in Figure 8a and b. The flexural stress and modulus generally increased with increasing laser power. For the samples printed at 15 and 18 W, the flexural stress shows significantly higher when built in X–Z compared with in X–Y.

The trend is similar to the previous literature reported on nylon-12 parts fabricated by PBF, where the flexural stress has shown to be the highest in X–Z orientation compared with X–Y.^35,36^ This indicates denser parts, which is the result of a higher concentration of the laser energy over a small surface area and laser path (as seen in Fig. 8c). Each layer of the sample printed in X–Z has more uniform density compared with each layer of the samples built in X–Y. The samples printed in X–Z orientation then have higher flexural stress and modulus than printed in X–Y.

In this work, the porosity ranges from 79% to 86%, with the highest compressive yield strength obtained being 4.1 MPa (yield strain of 64%). Compared with the literature data (summarized in Table 4), the porous PEEK structures obtained here are promising in compressive properties. Samples fabricated by compression molding using salt as a porogen with comparative porosities to the current study (84% tapped salt and 79% dry mixing salt) achieved compressive yield strength values of 1.22 MPa (tapped salt) and 1.85 MPa (dry mixing salt) and yield stain of ∼5%.^15^

**Table 4.** Summary of the Literature Data on Porous Poly (Ether-Ether-Ketone)

Reference	Methods	Compressive yield strength (MPa)	Compressive yield strain (%)	Porosity (%)	Pore size
This work	PBF + salt leaching	4.1	64	83.5	∼2–500 μm
Siddiq and Kennedy^15^	Compression molding of PEEK + salt leaching	1.85 (dry mixing)	∼5	79.2	0.54 mm
		1.22 (tapped salt)	∼5	83.9	0.58 mm
Vaezi and Yang^26^	FFF printing of PEEK	29.34	4.4	38	450 μm
Zheng *et al.*^25^	FFF printing of PEEK/HA	2.2–35.2	N/A	47.3–87.8	0.2–2.0 mm
Tan *et al.*^31,32^	PBF printing of PEEK + HA	N/A	N/A	73.5	N/A
Roskies *et al.*^5^	PBF printing of PEEK	N/A	N/A	36.38	N/A

FFF, fused filament fabrication; HA, hydroxyapatite; N/A, not applicable; PBF, powder bed fusion.

The porosity and compressive properties of the porous PEEK structure fabricated in this work are in the range of human trabecular bone (porosity: 74%–92%, compressive modulus: 0.3–3.2 GPa; and compressive yield strength: 0.7–17 MPa^35,36^); therefore, it is a promising structure for tissue engineering. Compared with the traditional preparation methods such as compression molding and extrusion processes, PBF overcomes limitations in design flexibility and customization, providing a possibility to fabricate multifunctional structures.^12,16^ Previous studies used AM techniques such as FFF and PBF to directly print porous structures, whereas here, the PBF fabrication method was combined with salt as a porogen, hence offering increased flexibility in pore sizes and overall porosity.^5,26,27,31,32^

## Conclusions

A salt and PEEK powder blend was designed to produce porous PEEK structures using the PBF process. This technique relies upon the removal of salt porogen through the generated open pores, forming a cellular PEEK structure with good mechanical properties. Cuboids with variable width from 10 × 1 × 10 mm to 10 × 10 × 10 mm, with an increase of 1 mm in width, were successfully built in two different orientations X–Y and X–Z. High porosity (up to 85.8%) with high compressive yield stress (4.1 MPa) of PEEK porous structure has been achieved in this study.

It was also noted that printing orientation and porosity were not the only factors affecting compressive properties. Cell wall thickness has also affected mechanical behavior. The flexural properties of porous PEEK structures were strongly affected by the printing orientation. This work demonstrated a novel technique to fabricate high-porosity structures with good mechanical performance. A better control of the morphology and particle sizes of the porogen as well as of the main polymer could lead to a more controlled, yet random pore size distribution, applicable to a broad range of sectors.

## Supplementary Material

Supplemental data

Supplemental data
