# Supplementary material for: Multiscale Porous Poly (Ether-Ether-Ketone) Structures Manufactured by Powder Bed Fusion Process
Source: 3D Print Addit Manuf. 2024 Feb 15;11(1):219–30. doi: 10.1089/3dp.2021.0317 (PMC10880674; doi:10.1089/3dp.2021.0317)
Supplement: Supplemental data [file Suppl_FigS1.docx]

Supplementary

Fabrication and characterization of porous poly (ether-ether-ketone) (PEEK) manufactured by powder bed fusion process

*Yaan Liu^1^, Richard Davies^1^, Nan Yi^1^, Paul McCutchion^1,^ Binling Chen^1^, Oana Ghita^1^*

***
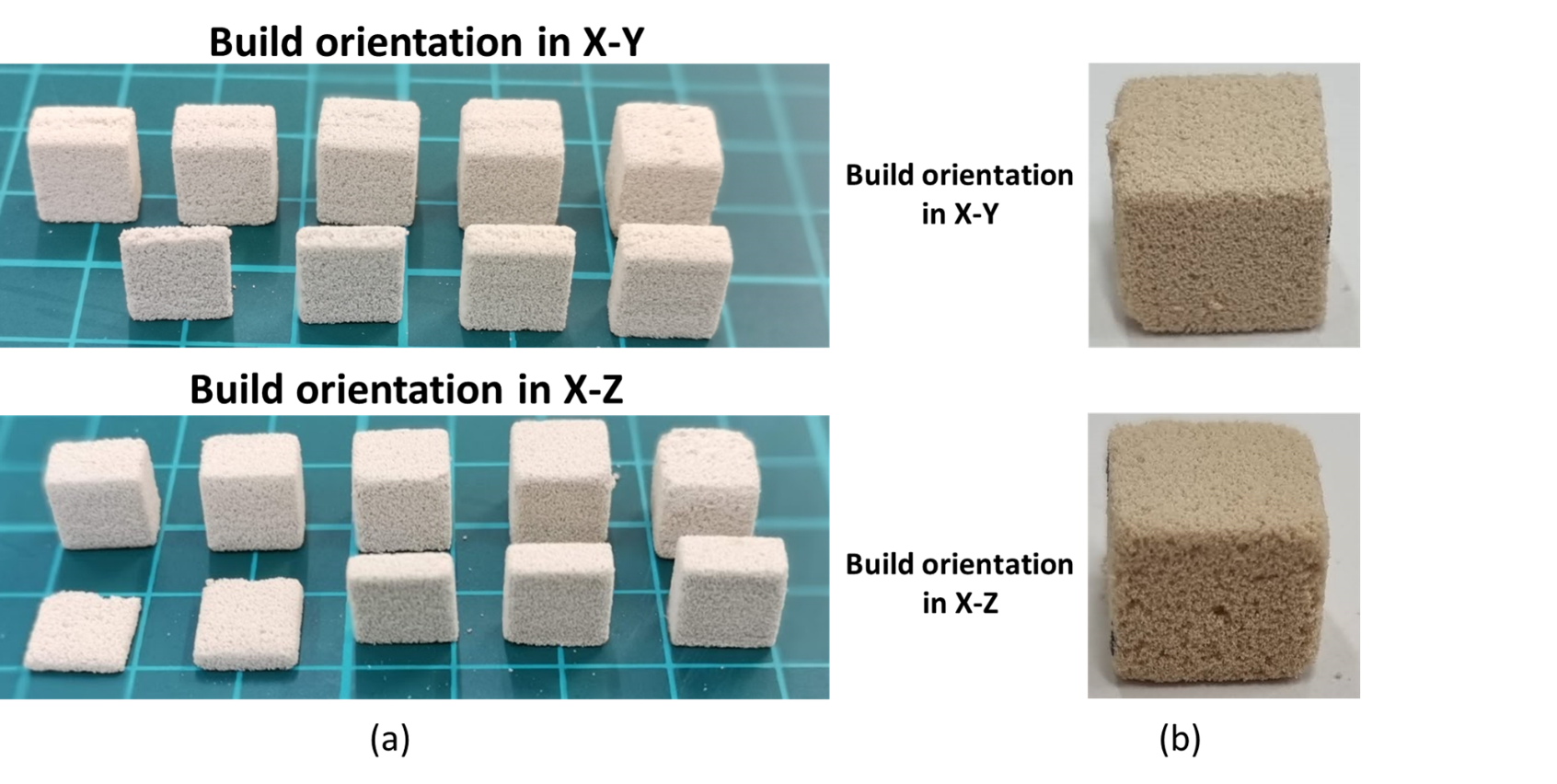
***

**Fig. S1.** (a)10 cuboids with constant length and height but variable width from 10$\times$1$\times$10 mm to 10$\times$10$\times$10 mm with an increase of 1 mm in width built in two different orientations X-Y and X-Z using laser powers of 18 W; (b) 10$\times$10$\times$10 mm cubic samples built in X-Y and X-Z using 18 W.
