# Supplementary material for: Multiscale Porous Poly (Ether-Ether-Ketone) Structures Manufactured by Powder Bed Fusion Process
Source: 3D Print Addit Manuf. 2024 Feb 15;11(1):219–30. doi: 10.1089/3dp.2021.0317 (PMC10880674; doi:10.1089/3dp.2021.0317)
Supplement: Supplemental data [file Suppl_TableS1.docx]

**Table S1** The size and weight changes of porous PEEK 450PF-T250_salt70 with variable width from 10$\times$1$\times$10 mm to 10$\times$10$\times$10 mm with an increase of 1 mm in width built in two different orientations X-Y and X-Z.

| Laser power and build orientation | Design size  (mm$\times$mm$\times$mm) | Dimension before salt leaching (mm$\times$mm$\times$mm) | Dimension after salt leaching  (mm$\times$mm$\times$mm) | Weight before salt leaching (g) | Weight after salt leaching (g) | Weight remained (%) |
| --- | --- | --- | --- | --- | --- | --- |
| 12W in X-Y | 10$\times$1$\times$10 | N/A | N/A | N/A | N/A | N/A |
|  | 10$\times$2$\times$10 | 10.33$\times$1.71$\times$9.39 | 10.03$\times$9.0.$\times$1.61 | 0.115 | 0.031 | 26.56 |
|  | 10$\times$3$\times$10 | 10.10$\times$2.78$\times$9.54 | 9.95$\times$9.73$\times$2.66 | 0.188 | 0.055 | 29.23 |
|  | 10$\times$4$\times$10 | 10.25$\times$3.76$\times$9.60 | 10.07$\times$9.47$\times$3.63 | 0.254 | 0.076 | 29.80 |
|  | 10$\times$5$\times$10 | 10.38$\times$4.73$\times$9.57 | 10.17$\times$9.26$\times$4.52 | 0.320 | 0.095 | 29.60 |
|  | 10$\times$6$\times$10 | 10.39$\times$5.65$\times$9.58 | 10.11$\times$9.40$\times$5.49 | 0.387 | 0.115 | 29.62 |
|  | 10$\times$7$\times$10 | 10.27$\times$6.58$\times$9.46 | 10.23$\times$9.30$\times$6.41 | 0.449 | 0.135 | 30.01 |
|  | 10$\times$8$\times$10 | 10.49$\times$7.52$\times$9.50 | 10.18$\times$9.25$\times$7.38 | 0.521 | 0.158 | 30.37 |
|  | 10$\times$9$\times$10 | 10.36$\times$8.52$\times$9.49 | 10.24$\times$9.39$\times$8.43 | 0.584 | 0.182 | 31.21 |
|  | 10$\times$10$\times$10 | 10.45$\times$9.52$\times$9.51 | 10.26$\times$9.34$\times$9.56 | 0.650 | 0.200 | 30.77 |
| 13.5W in X-Y | 10$\times$1$\times$10 | N/A | N/A | N/A | N/A | N/A |
|  | 10$\times$2$\times$10 | 10.24$\times$1.88$\times$9.63 | 9.55$\times$1.82$\times$9.98 | 0.126 | 0.033 | 26.53 |
|  | 10$\times$3$\times$10 | 9.74$\times$2.88$\times$9.69 | 9.37$\times$2.62$\times$9.37 | 0.187 | 0.051 | 27.47 |
|  | 10$\times$4$\times$10 | 10.24$\times$3.95$\times$9.75 | 9.79$\times$3.65$\times$9.45 | 0.266 | 0.078 | 29.17 |
|  | 10$\times$5$\times$10 | 10.23$\times$4.85$\times$9.65 | 9.88$\times$4.51$\times$9.37 | 0.334 | 0.098 | 29.40 |
|  | 10$\times$6$\times$10 | 10.52$\times$5.82$\times$9.69 | 10.22$\times$5.52$\times$9.31 | 0.404 | 0.120 | 29.59 |
|  | 10$\times$7$\times$10 | 10.37$\times$4.72$\times$9.65 | 10.19$\times$6.49$\times$9.37 | 0.468 | 0.138 | 29.42 |
|  | 10$\times$8$\times$10 | 10.44$\times$7.67$\times$9.63 | 10.15$\times$7.55$\times$9.40 | 0.536 | 0.162 | 30.21 |
|  | 10$\times$9$\times$10 | 10.37$\times$8.64$\times$9.68 | 10.14$\times$8.42$\times$9.48 | 0.608 | 0.187 | 30.71 |
|  | 10$\times$10$\times$10 | 10.53$\times$9.64$\times$9.64 | 10.14$\times$9.39$\times$9.40 | 0.670 | 0.210 | 31.34 |
| 15W in X-Y | 10$\times$1$\times$10 | N/A | N/A | N/A | N/A | N/A |
|  | 10$\times$2$\times$10 | 10.41$\times$1.87$\times$9.59 | 9.88$\times$1.77$\times$9.44 | 0.129 | 0.036 | 27.82 |
|  | 10$\times$3$\times$10 | 10.20$\times$2.91$\times$9.68 | 9.46$\times$2.69$\times$9.95 | 0.199 | 0.057 | 28.54 |
|  | 10$\times$4$\times$10 | 10.43$\times$3.95$\times$9.74 | 10.17$\times$3.75$\times$9.50 | 0.268 | 0.079 | 29.46 |
|  | 10$\times$5$\times$10 | 10.49$\times$4.88$\times$9.82 | 10.14$\times$4.64$\times$9.52 | 0.340 | 0.100 | 29.41 |
|  | 10$\times$6$\times$10 | 10.31$\times$5.82$\times$9.80 | 10.37$\times$5.62$\times$9.52 | 0.410 | 0.122 | 29.78 |
|  | 10$\times$7$\times$10 | 10.44$\times$6.73$\times$9.70 | 10.09$\times$6.59$\times$9.52 | 0.476 | 0.145 | 30.39 |
|  | 10$\times$8$\times$10 | 10.48$\times$7.75$\times$9.69 | 10.29$\times$7.55$\times$9.48 | 0.553 | 0.172 | 31.13 |
|  | 10$\times$9$\times$10 | 10.43$\times$8.67$\times$9.67 | 10.62$\times$8.54$\times$9.48 | 0.621 | 0.202 | 32.49 |
|  | 10$\times$10$\times$10 | 10.58$\times$9.70$\times$9.69 | 10.23$\times$9.40$\times$9.27 | 0.690 | 0.200 | 28.99 |
| 16.5W in X-Y | 10$\times$1$\times$10 | N/A | N/A | N/A | N/A | N/A |
|  | 10$\times$2$\times$10 | 9.90$\times$1.80$\times$9.65 | 9.70$\times$1.74$\times$9.43 | 0.123 | 0.035 | 28.04 |
|  | 10$\times$3$\times$10 | 10.03$\times$2.94$\times$9.59 | 9.65$\times$2.77$\times$9.45 | 0.196 | 0.056 | 28.74 |
|  | 10$\times$4$\times$10 | 10.43$\times$3.93$\times$9.81 | 10.00$\times$3.63$\times$9.28 | 0.274 | 0.081 | 29.51 |
|  | 10$\times$5$\times$10 | 10.48$\times$4.91$\times$9.81 | 10.05$\times$4.60$\times$9.47 | 0.347 | 0.104 | 29.92 |
|  | 10$\times$6$\times$10 | 10.15$\times$5.96$\times$9.77 | 9.50$\times$5.70$\times$9.65 | 0.400 | 0.120 | 30.02 |
|  | 10$\times$7$\times$10 | 10.62$\times$6.78$\times$9.68 | 10.37$\times$6.60$\times$9.46 | 0.483 | 0.150 | 31.01 |
|  | 10$\times$8$\times$10 | 10.73$\times$7.74$\times$9.74 | 10.29$\times$7.67$\times$9.48 | 0.551 | 0.178 | 32.37 |
|  | 10$\times$9$\times$10 | 10.53$\times$8.70$\times$9.66 | 10.34$\times$8.49$\times$9.43 | 0.624 | 0.205 | 32.86 |
|  | 10$\times$10$\times$10 | 10.77$\times$9.63$\times$9.82 | 10.14$\times$9.18$\times$9.21 | 0.690 | 0.200 | 28.99 |
| 18W in X-Y | 10$\times$1$\times$10 | N/A | N/A | N/A | N/A | N/A |
|  | 10$\times$2$\times$10 | 10.04$\times$1.99$\times$9.79 | 9.69$\times$1.85$\times$9.29 | 0.134 | 0.039 | 29.38 |
|  | 10$\times$3$\times$10 | 10.43$\times$2.99$\times$9.84 | 10.10$\times$2.78$\times$9.57 | 0.210 | 0.063 | 29.95 |
|  | 10$\times$4$\times$10 | 10.40$\times$3.97$\times$9.81 | 10.34$\times$3.95$\times$9.63 | 0.282 | 0.095 | 33.77 |
|  | 10$\times$5$\times$10 | 10.58$\times$4.93$\times$9.85 | 10.56$\times$4.94$\times$9.66 | 0.358 | 0.135 | 37.70 |
|  | 10$\times$6$\times$10 | 10.60$\times$5.88$\times$9.87 | 10.53$\times$5.91$\times$9.72 | 0.436 | 0.176 | 40.42 |
|  | 10$\times$7$\times$10 | 10.62$\times$6.86$\times$9.82 | 10.52$\times$6.77$\times$9.62 | 0.508 | 0.166 | 32.59 |
|  | 10$\times$8$\times$10 | 10.90$\times$7.82$\times$9.84 | 10.62$\times$7.67$\times$9.63 | 0.585 | 0.196 | 33.44 |
|  | 10$\times$9$\times$10 | 10.78$\times$8.75$\times$9.84 | 10.34$\times$8.58$\times$9.63 | 0.653 | 0.226 | 34.57 |
|  | 10$\times$10$\times$10 | 10.84$\times$9.76$\times$9.76 | 10.51$\times$9.60$\times$9.62 | 0.740 | 0.240 | 32.43 |
| 12W in X-Z | 10$\times$1$\times$10 | 9.32$\times$1.53$\times$9.41 | 9.30$\times$1.31$\times$9.02 | 0.084 | 0.024 | 29.18 |
|  | 10$\times$2$\times$10 | 9.48$\times$2.44$\times$9.44 | 9.37$\times$2.44$\times$9.37 | 0.146 | 0.043 | 29.49 |
|  | 10$\times$3$\times$10 | 9.60$\times$3.50$\times$9.82 | 9.63$\times$3.26$\times$9.32 | 0.217 | 0.062 | 28.61 |
|  | 10$\times$4$\times$10 | 9.81$\times$4.43$\times$9.65 | 9.50$\times$4.13$\times$9.33 | 0.277 | 0.085 | 30.60 |
|  | 10$\times$5$\times$10 | 9.78$\times$5.38$\times$9.62 | 9.26$\times$5.27$\times$9.34 | 0.337 | 0.104 | 31.02 |
|  | 10$\times$6$\times$10 | 9.59$\times$6.44$\times$9.91 | 9.30$\times$6.23$\times$9.50 | 0.402 | 0.124 | 30.98 |
|  | 10$\times$7$\times$10 | 9.51$\times$7.35$\times$9.58 | 9.36$\times$7.14$\times$9.28 | 0.463 | 0.146 | 31.62 |
|  | 10$\times$8$\times$10 | 9.52$\times$8.25$\times$9.58 | 9.32$\times$8.16$\times$9.23 | 0.521 | 0.166 | 31.90 |
|  | 10$\times$9$\times$10 | 9.52$\times$9.30$\times$9.69 | 9.45$\times$9.07$\times$9.36 | 0.581 | 0.189 | 32.55 |
|  | 10$\times$10$\times$10 | 9.60$\times$10.21$\times$9.63 | 9.40$\times$10.14$\times$9.45 | 0.640 | 0.190 | 29.69 |
| 13.5W in X-Z | 10$\times$1$\times$10 | 9.58$\times$1.59$\times$9.48 | 9.50$\times$1.47$\times$9.42 | 0.091 | 0.028 | 30.77 |
|  | 10$\times$2$\times$10 | 9.69$\times$2.60$\times$10.27 | 9.39$\times$2.60$\times$10.12 | 0.172 | 0.050 | 28.83 |
|  | 10$\times$3$\times$10 | 9.85$\times$3.55$\times$9.69 | 9.41$\times$3.37$\times$9.42 | 0.225 | 0.064 | 28.53 |
|  | 10$\times$4$\times$10 | 9.82$\times$4.52$\times$9.84 | 9.53$\times$4.26$\times$9.53 | 0.285 | 0.082 | 28.65 |
|  | 10$\times$5$\times$10 | 9.80$\times$5.41$\times$9.96 | 9.42$\times$5.21$\times$9.42 | 0.347 | 0.098 | 28.10 |
|  | 10$\times$6$\times$10 | 9.76$\times$6.53$\times$9.83 | 9.46$\times$6.29$\times$9.46 | 0.412 | 0.121 | 29.40 |
|  | 10$\times$7$\times$10 | 9.71$\times$7.48$\times$9.66 | 9.43$\times$7.25$\times$9.43 | 0.478 | 0.144 | 30.20 |
|  | 10$\times$8$\times$10 | 9.73$\times$8.42$\times$9.65 | 9.48$\times$8.20$\times$9.48 | 0.547 | 0.172 | 31.37 |
|  | 10$\times$9$\times$10 | 9.70$\times$9.38$\times$9.71 | 9.54$\times$9.19$\times$9.54 | 0.613 | 0.199 | 32.45 |
|  | 10$\times$10$\times$10 | 9.72$\times$10.31$\times$9.65 | 9.42$\times$10.14$\times$9.42 | 0.680 | 0.210 | 30.88 |
| 15W in X-Z | 10$\times$1$\times$10 | 9.53$\times$1.63$\times$9.17 | 9.34$\times$1.36$\times$9.14 | 0.094 | 0.026 | 27.48 |
|  | 10$\times$2$\times$10 | 9.67$\times$2.62$\times$10.08 | 9.42$\times$2.36$\times$9.79 | 0.170 | 0.049 | 29.05 |
|  | 10$\times$3$\times$10 | 9.78$\times$3.50$\times$9.98 | 9.46$\times$3.35$\times$9.68 | 0.235 | 0.067 | 28.43 |
|  | 10$\times$4$\times$10 | 9.72$\times$4.59$\times$9.79 | 9.42$\times$4.32$\times$9.48 | 0.315 | 0.087 | 27.58 |
|  | 10$\times$5$\times$10 | 9.86$\times$5.57$\times$9.86 | 9.52$\times$5.42$\times$9.55 | 0.354 | 0.110 | 31.09 |
|  | 10$\times$6$\times$10 | 9.75$\times$6.50$\times$9.93 | 9.37$\times$6.41$\times$9.68 | 0.427 | 0.136 | 31.81 |
|  | 10$\times$7$\times$10 | 9.72$\times$7.51$\times$9.77 | 9.44$\times$7.32$\times$9.57 | 0.488 | 0.161 | 33.00 |
|  | 10$\times$8$\times$10 | 9.77$\times$8.43$\times$9.66 | 9.61$\times$8.29$\times$9.50 | 0.552 | 0.185 | 33.47 |
|  | 10$\times$9$\times$10 | 9.81$\times$9.94$\times$9.71 | 9.47$\times$9.07$\times$9.41 | 0.620 | 0.210 | 33.92 |
|  | 10$\times$10$\times$10 | 9.69$\times$10.51$\times$9.75 | 9.40$\times$10.12$\times$9.33 | 0.100 | 0.190 | 27.94 |
| 16.5W in X-Z | 10$\times$1$\times$10 | 9.50$\times$1.68$\times$9.49 | 9.05$\times$1.53$\times$9.41 | 0.172 | 0.030 | 30.25 |
|  | 10$\times$2$\times$10 | 9.67$\times$2.66$\times$9.74 | 9.59$\times$2.50$\times$9.46 | 0.247 | 0.054 | 31.57 |
|  | 10$\times$3$\times$10 | 9.75$\times$3.62$\times$9.88 | 9.44$\times$3.32$\times$9.59 | 0.309 | 0.072 | 29.01 |
|  | 10$\times$4$\times$10 | 9.83$\times$4.55$\times$9.86 | 9.55$\times$4.37$\times$9.53 | 0.368 | 0.091 | 29.42 |
|  | 10$\times$5$\times$10 | 9.87$\times$5.58$\times$9.91 | 9.48$\times$5.48$\times$9.68 | 0.443 | 0.116 | 31.62 |
|  | 10$\times$6$\times$10 | 10.04$\times$6.54$\times$9.79 | 9.58$\times$6.41$\times$9.68 | 0.510 | 0.146 | 32.93 |
|  | 10$\times$7$\times$10 | 9.97$\times$7.57$\times$10.04 | 9.59$\times$7.48$\times$9.88 | 0.636 | 0.173 | 33.92 |
|  | 10$\times$8$\times$10 | 9.71$\times$8.48$\times$9.79 | 9.56$\times$8.31$\times$9.61 | 0.635 | 0.197 | 31.03 |
|  | 10$\times$9$\times$10 | 9.64$\times$9.62$\times$9.79 | 9.44$\times$9.24$\times$9.53 | 0.690 | 0.227 | 35.82 |
|  | 10$\times$10$\times$10 | 9.64$\times$10.40$\times$9.91 | 9.32$\times$10.01$\times$9.30 | 0.100 | 0.210 | 30.43 |
| 18W in X-Z | 10$\times$1$\times$10 | 9.50$\times$1.86$\times$9.64 | 9.47$\times$1.65$\times$9.47 | 0.114 | 0.036 | 31.81 |
|  | 10$\times$2$\times$10 | 9.93$\times$2.79$\times$10.03 | 9.76$\times$2.58$\times$9.76 | 0.192 | 0.061 | 31.73 |
|  | 10$\times$3$\times$10 | 10.16$\times$3.79$\times$10.00 | 9.86$\times$3.58$\times$9.86 | 0.257 | 0.079 | 30.96 |
|  | 10$\times$4$\times$10 | 9.89$\times$4.63$\times$9.68 | 9.50$\times$4.55$\times$9.50 | 0.303 | 0.098 | 32.26 |
|  | 10$\times$5$\times$10 | 10.03$\times$5.70$\times$10.08 | 9.87$\times$5.49$\times$9.87 | 0.388 | 0.127 | 32.72 |
|  | 10$\times$6$\times$10 | 9.92$\times$6.71$\times$10.19 | 9.81$\times$6.47$\times$9.81 | 0.462 | 0.159 | 34.27 |
|  | 10$\times$7$\times$10 | 9.87$\times$7.74$\times$9.95 | 9.82$\times$7.46$\times$9.82 | 0.535 | 0.189 | 35.35 |
|  | 10$\times$8$\times$10 | 10.02$\times$8.54$\times$9.98 | 9.99$\times$8.41$\times$9.99 | 0.600 | 0.215 | 35.88 |
|  | 10$\times$9$\times$10 | 9.83$\times$9.48$\times$9.86 | 9.77$\times$9.34$\times$9.77 | 0.663 | 0.247 | 37.22 |
|  | 10$\times$10$\times$10 | 9.81$\times$10.46$\times$9.94 | 9.74$\times$10.13$\times$9.74 | 0.710 | 0.220 | 30.99 |
